# Matrilin-2 within a three-dimensional lysine-modified chitosan porous scaffold enhances Schwann cell migration and axonal outgrowth for peripheral nerve regeneration

**DOI:** 10.3389/fbioe.2023.1142610

**Published:** 2023-05-04

**Authors:** Neill Y. Li, Brandon Vorrius, Jonathan Ge, Zhen Qiao, Shuang Zhu, Julia Katarincic, Qian Chen

**Affiliations:** ^1^ Laboratory of Molecular Biology and Nanomedicine, Department of Orthopaedics, Warren Alpert Medical School of Brown University, Providence, RI, United States; ^2^ Department of Orthopaedics, Duke University School of Medicine, Durham, NC, United States

**Keywords:** nerve regeneration, scaffold, chitosan, matrilin-2, schwann cell, axon, porous, lysine

## Abstract

**Background:** Matrilin-2 is a key extracellular matrix protein involved in peripheral nerve regeneration. We sought to develop a biomimetic scaffold to enhance peripheral nerve regeneration by incorporating matrilin-2 within a chitosan-derived porous scaffold. We hypothesized that the use of such a novel biomaterial delivers microenvironmental cues to facilitate Schwann cell (SC) migration and enhance axonal outgrowth during peripheral nerve regeneration.

**Materials and Methods:** The effect of matrilin-2 on SC migration was evaluated with agarose drop migration assay on matrilin-2 coated dishes. SC adhesion was determined with SCs cultured atop tissue culture dishes coated with matrilin-2. Various formulations of chitosan vs matrilin-2 in scaffold constructs were examined with scanning electron microscopy. The effect of the matrilin-2/chitosan scaffold on SC migration in the collagen conduits was determined by capillary migration assays. Neuronal adhesion and axonal outgrowth were evaluated with three-dimensional (3D) organotypic assay of dorsal root ganglions (DRG). DRG axonal outgrowth within the scaffolds was determined by immunofluorescence staining of neurofilaments.

**Results:** Matrilin-2 induced SC migration and enhanced its adhesion. A formulation of 2% chitosan with matrilin-2 demonstrated an optimal 3D porous architecture for SC interaction. Matrilin-2/chitosan scaffold enabled SCs to migrate against gravity within conduits. Chemical modification of chitosan with lysine (K-chitosan) further improved DRG adhesion and axonal outgrowth than the matrilin-2/chitosan scaffold without lysine modification.

**Conclusion:** We developed a matrilin-2/K-chitosan scaffold to mimic extracellular matrix cues and provide a porous matrix to enhance peripheral nerve regeneration. Taking advantage of matrilin-2’s capability to stimulate SC migration and adhesion, we formulated a porous matrilin-2/chitosan scaffold to support axongal outgrowth. Chemical modification of chitosan with lysine further improved matrilin-2 bioactivity in the 3D scaffold. The 3D porous matrilin-2/K-chitosan scaffolds have high potential for enhancing nerve repair by stimulating SC migration, neuronal adhesion, and axonal outgrowth.

## Introduction

Despite adequate management, patients with major peripheral nerve injuries (PNI) undertake a long recovery to regain motor and sensory function. As a result, the potential and permanence for a significant reduction in quality of life and independence are high. PNI may occur following motor vehicle accidents, penetrating wounds, or crush injuries leaving the nerve lacerated, compressed, stretched, or avulsed (Rotshenker, 2015). Depending on the state and chronicity of the injury, the nerve may not be amenable to repair by primary end-to-end coaptation. If there is nerve loss or need for excision in which direct repair would cause excessive tension, means to reconstruct the gap through artificial conduit, acellular nerve allograft (ANA), or autograft may be utilized depending on the distance to reconstruct.

Currently, the use of conduits *versus* allograft or autograft depends on the extent of nerve injury and the length of gapping between nerve ends that require repair (Ducic et al., 2012). Nerve conduits have been produced from a variety of materials including type I collagen, polyglycolic acid, porcine small intestine submucosa, and chitosan (Zhang et al., 2021; Zhang et al., 2022; Wang et al., 2023). The tubular and hollow structure may restrict the use of conduits to short gaps of around 5 mm with a maximum of less than 10 mm in small nerve diameters as their construction lacks the internal architecture to support more considerable cross-sectional regeneration and distances (Shin et al., 2009; Giusti et al., 2012; Lin et al., 2013). For longer gaps, ANAs have become more commonly used. ANAs provide the native architecture and platform for regeneration, but the processing and use of detergents to remove cells, extracellular matrix (ECM) proteins, and growth factors conducive to nerve regeneration, limit the length and robustness of regeneration that become more suited for nerve autograft. Autografts are the gold standard for bridging gaps in PNI. The use of autograft provides the native architecture and proteins that critically form the ECM as well as growth factors to facilitate the best microenvironment for regeneration. However, harvesting nerve from another part of the body can lead to adverse effects such as incision site complications, neuromas at the repair site, and loss of sensory function (Giusti et al., 2012).

Given the positives and negatives of each modality, we sought to construct a readily available graft that features a biomimetic scaffold within a multichannel three-dimensional cross-sectional architecture inherent to nerves to bridge longer gaps than currently used conduits to achieve a more robust regenerative response. To initiate a biomimetic environment, we sought to exploit the properties of the ECM protein family of matrilins (Wang et al., 2014). In nerve, matrilin-2 is expressed in the perineurium and upregulated by Schwann cells (SCs) following injury (Korpos et al., 2015). Matrilin-2 belongs to an oligomeric multi-adhesion adaptor protein family named matrilins with a wide distribution in the ECM of different tissues. They interact among various ECM ligands including collagen, fibrillin, laminin, and fibronectin (Piecha et al., 1999). Such interactions are mediated by the metal-ion-dependent adhesion site (MIDAS) (Kim et al., 2016). Notably, the lysine residue is often involved in the interaction of ECM ligands (Mäki et al., 2005). The broad distribution of matrilin-2 allows it to mediate ECM protein adhesion and assembly (Segat et al., 2000; Piecha et al., 2002). Matrilin-2 enhances axonal outgrowth and SC migration upon its application *in-vitro* and *ex-vivo* and further verified in MATN2 knockout mouse models (Malin et al., 2009).

The aim of this study is to utilize matrilin-2 in a reconstructive environment for nerve repair. We hypothesized that increased cross-sectional and longitudinal presence of matrilin-2 improves the ability for SCs to adhere and migrate, thereby enhancing the rate of axonal outgrowth and ultimately neural regeneration. To test this hypothesis, we first examined whether matrilin-2 maintained chemotactic properties of SC migration *in vitro*. We then developed a scaffold with the cross-sectional architecture that hosts matrilin-2 utilizing material well suited in a neural regenerative environment. Utilizing iterations of the scaffold, we demonstrate that axonal outgrowth of neonatal dorsal root ganglions (DRG) was improved compared to currently used conduits. We further identified that chemical modification of chitosan with lysine further improved neuronal adhesion, SC migration, and axonal outgrowth by enhancing matrilin-2 conjugation to form matrilin-2/K-chitosan scaffold.

## Materials and methods

### Cell culture

S16 SCs were purchased from American Type Culture Collection (ATCC, Manassas, VA) and were cultured per ATCC protocol in DMEM/F-12 supplemented with 10% FBS and 1% Penicillin-Streptomycin. All media and supplements were purchased from Thermo Fisher Scientific (Waltham, MA).

### Agarose migration and cell adhesion assays

Recombinant human matrilin-2 protein was purchased from R&D systems (Minneapolis, MN), coated onto 22 mm tissue glass coverslips at 20ug/ml as described previously (Malin et al., 2009) and incubated overnight. The agarose migration assay was performed as previously described (Chernousov et al., 2001). The agarose for the drop migration assay was diluted to a 0.03% concentration. After 24 h of incubation, the cells were visualized under phase-contrast microscopy.

For cellular adhesion, SCs were placed atop matrilin-2 coated plates and incubated for 24 h. Cells were then fixed at room temperature with 4% paraformaldehyde for 10 min. Subsequently, cells were washed with PBS, followed by permeabilization for 30 min using 0.1% Triton X-100. Cells were stained with rhodamine phalloidin (Cell Signaling Technology, Danvers, MA) for immunofluorescent imaging. After 30 min, the cells were washed three times with PBS and incubated in 4′,6′-diamidino-2-phenylindole dihydrochloride (1:1,000; Sigma-Aldrich, St. Louis, MO) for 5 min at room temperature to counterstain the cell nuclei. The specimens were washed three times with PBS, each for 5 min, and were then mounted with an antifade solution. Immunolabeled cells were examined *via* fluorescence microscopy (Zeiss Axiovision Z1, Oberkochen, Germany). All experiments were carried out in triplicate.

### Material survey and evaluation

**FIGURE 1:**
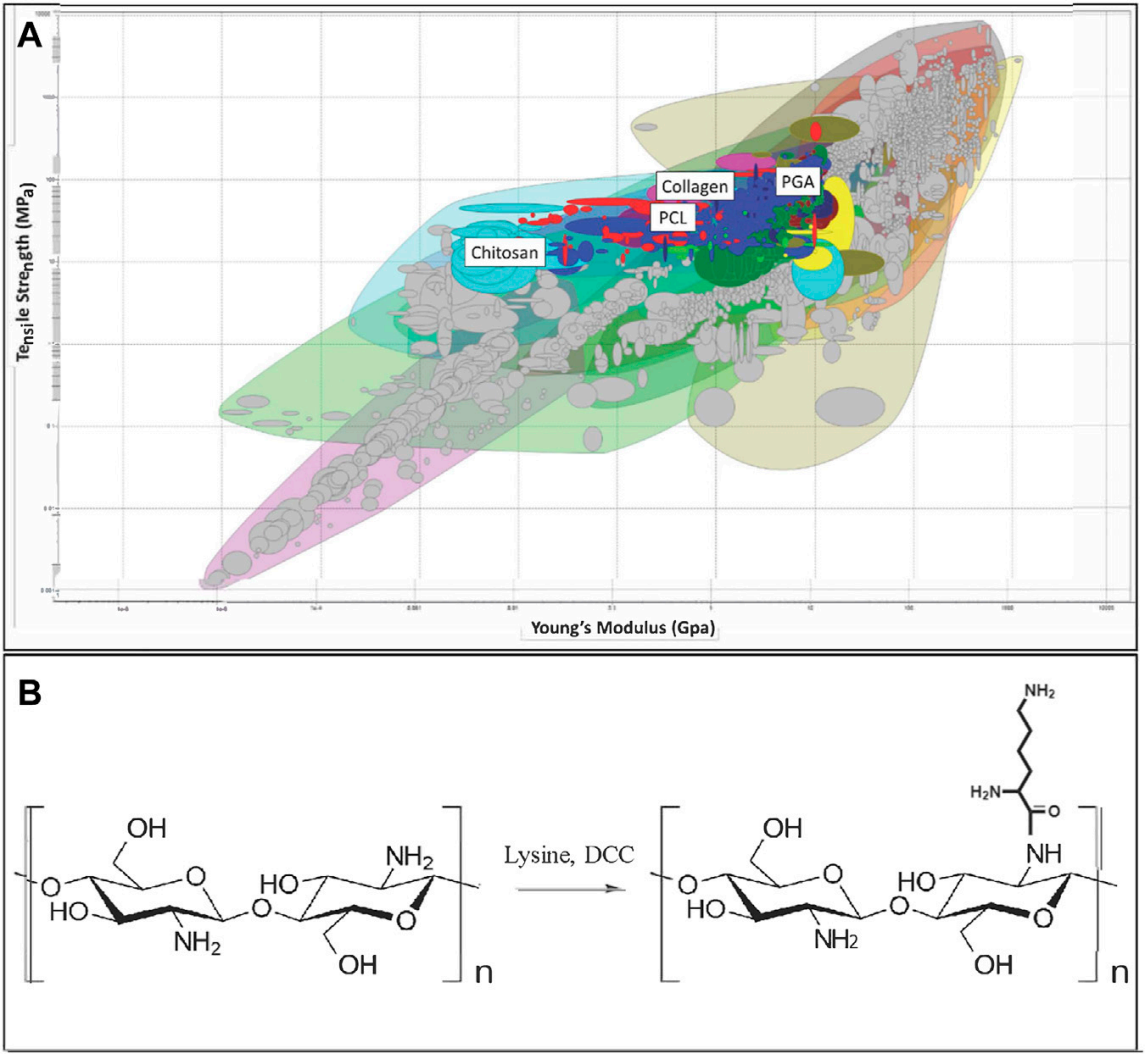
**(A)** Initial array of materials with tensile strength and Young’s Modulus as constraints. Materials highlighted include Polyglycolic Acid (PGA), Polycaprolactone (PCL), collagen, and chitosan. **(B)** Chitosan structure (Left) and chemical modification of chitosan by Lysine (K-chitosan).

Given the wide variety of materials previously utilized for nerve repair, a material selection search was performed to determine an ideal material to interact with matrilin-2 in the formation of a scaffold. The CES Edupack Database of Natural and Man-Made materials (Ansys, Canonsburg, PA) was utilized to comprehensively examine available options. The database was used with an initial filter of material of tensile strength of 12–100 Megapascals. The following parameter was Young’s Modulus with biological reference to 0.067 GPa for peripheral nerves (Borschel et al., 2003). This was set to a constraint of 0.005–10 Gigapascals (GPa). This range included polymer-based rubbers as a point of interest as well as chitosan (0.014 GPa), which has the lowest Young’s Modulus of the currently used materials in nerve conduits. The upper constraint of the modulus was set to 10.0 GPa to include a large variance of potential materials. PGA had the highest Young’s Modulus of currently used materials with 6.1–7.2 GPa (Figure 1A).

### Scaffold formulation with Matrilin-2 and chitosan

Unless otherwise specified, all chemicals were obtained from Sigma-Aldrich (St. Louis, MO). Chitosan was dissolved in acetic acid in the development of various concentrations of chitosan at 1%, 2%, and 3%. Matrilin-2 was then added within a physiologic concentration level of 10 ug/mL as exemplified by Malin et al. (Jonas et al., 2014). 50uL of solutions were then pipetted into 2 mm × 2 mm sectioned pieces of a collagen conduit (Integra, Princeton, NJ). These units were then flash-frozen and lyophilized. Scaffolds were then fixed and imaged under scanning electron microscopy to detail their microarchitecture.

### Capillary migration study

S16 SCs were cultured as described above and stained with Vybrant Dil Cell-Labeling Solution (ThermoFisher Scientific, Waltham, MA). The cells were then seeded into a 24-well cell culture plate. A 5 mm long empty or scaffold-filled conduit was then placed vertically atop the cells, in which the cross-section of the conduit faced the cells. SCs were incubated for 24 h to allow for migration up the conduit and/or scaffold. The conduits were then longitudinally sectioned and fixed onto slides to visualize the SC migration under fluorescence.

### Chitosan modification with lysine

To improve upon neuronal and SC interaction with matrilin-2 given poor adherence of DRGs during the 3D organotypic assay, chitosan was modified with lysine to enhance conjugation with matrilin-2 (Figure 1B). 100ml of 2% (w/v) chitosan aqueous solution was prepared with the addition of 1% (v/v%) acetic acid solution under vigorous mechanical stirring at room temperature to form homogeneous gel. Lysine was added at a lower molar ratio (0.1mM, 0.0146 g) than the free amine groups on the chitosan chain. The solution was stirred for 1 h to again form a homogeneous gel. Afterwards, 0.1mMol (0.0206 g) DCC (N,N′-Dicyclohexylcarbodiimide), dissolved in 150 μl 99% ethanol, was added to trigger the coupling reaction of chitosan and lysine. This reaction was carried out in room temperature, with gentle agitation for 1 h. Upon reaction completion, the gel was filtered to remove dicyclohexylurea (DCU). For crosslinking of lysine-modified chitosan (K-chitosan), 100 μl glutaraldehyde (25% in H_2_O) was added dropwise into the gel solution under vigorous stirring. The pH of the above system was then adjusted to 10.0 using NaOH (10% w/v) to instigate a crosslinking reaction for 2 h at 60 °C. The gel was then filtered and purified with DI-H2O in a dialysis bag for 4 h to remove unreacted chemicals and side products (trace amount DCU *etc.*). Matrilin-2 was then added in a similar fashion as described above to construct the matrilin-2 conjugated K-chitosan scaffold (matrilin-2/K-chitosan).

### Three-dimensional organotypic assay for DRG-Based axonal outgrowth

The assay was performed as described by (Tajdaran et al., 2019). Rat DRGs were purchased from BrainBits LLC (Springfield, IL). 2mm diameter collagen conduits were sectioned into 5 mm lengths. The conduits were separated into three groups: 1) collagen conduit with chitosan scaffold only, 2) conduit with matrilin-2/chitosan scaffold, and 3) conduit with matrilin-2/K-chitosan scaffold. Each conduit was placed separately into a 9-well tissue culture dish with each filled with media composed of neurobasal media, B27 supplement at 50X, NGF at 50 ng/ml, 1% Pencillin/Streptomycin, and Glutamax 100X. The conduits and associated scaffolds were allowed to incubate in the neurobasal media for 4 h at 37C. DRGs were then placed into each conduit or conduit and scaffold and allowed to incubate for 48 h at 37C.

After incubation, media was removed from the wells and the conduits were rinsed with PBS x 5 min, three times. Paraformaldehyde 4% was then used to fix the DRG and conduits overnight. The DRG and conduits were then rinsed with PBS x 5 min, 3 times, and then placed in incremental increases of sucrose starting with 10%–20% to 30% which was soaked overnight. The samples were then placed into trays and frozen individually in O.C.T compound embedding medium. The samples were cryosectioned longitudinally at the 200, 400, 600, 800, 1,000, 1,200, 1,400, 1,600, and 1800 μm levels and cut at 10 μm thickness. The collected sections were then processed for H&E and immunofluorescence staining. For immunofluorescence analysis, sections were washed, blocked (5% normal goat serum), and incubated with Anti-Neurofilament-L mouse monoclonal antibody (1:50, Santa Cruz) overnight at 4C. The sections were then washed and incubated with donkey anti-mouse Alexa Fluor 568 (1:200, In) for 2 h at room temperature. Sections were then washed and visualized under fluorescent microscopy.

## Results

### Selection of chitosan as a structural scaffold

We selected polymer candidates for a nerve regeneration scaffold by considering material properties as described in Methods and Materials (Figure 1A). We set the minimum tensile strength to 12 MPa and eliminated materials lacking appropriate elasticity according to the Young’s Modulus (Supplementary Figure S1). PGA, one of the previously described materials utilized for nerve conduits, was eliminated according to these criteria (Figure 1A). Next, by applying the criterion “Biological and natural materials,” the search was refined to include only chitosan, collagen, and collagen-related natural materials leather, and ligament (Supplementary Figure S1). This refinement was made because the biocompatibility of these materials would be important to interact with extracellular matrix proteins, growth factors, and chemokines, while also permitting cellular adherence.

We ultimately chose chitosan as the scaffold-forming material, taking into consideration that chitosan has a lower Young’s Modulus than that of collagen and peripheral nerve. This property could be beneficial as chitosan poses less resistance to stretching and yet maintains the tensile strength of a normal nerve. Such material property not only reduces the likelihood of material tearing during manufacturing, but also allows SCs to migrate and guide axonal regeneration during movement at the reconstruction site. We expect that as the SCs produce matrix during the regenerative process, the Young’s Modulus would be increased to approximate that of a healthy nerve (Borschel et al., 2003). Thus, we selected chitosan as the primary structural component of the scaffold within a collagen conduit. Previous tissue engineering research found success with chitosan and collagen interaction to improve mechanical strength, biodegradation rate, and cell proliferation, which are crucial for optimal axonal regeneration (Ma et al., 2003; Tangsadthakun et al., 2006).

### Matrilin-2 increased Schwann cell migration and adhesion

**FIGURE 2:**
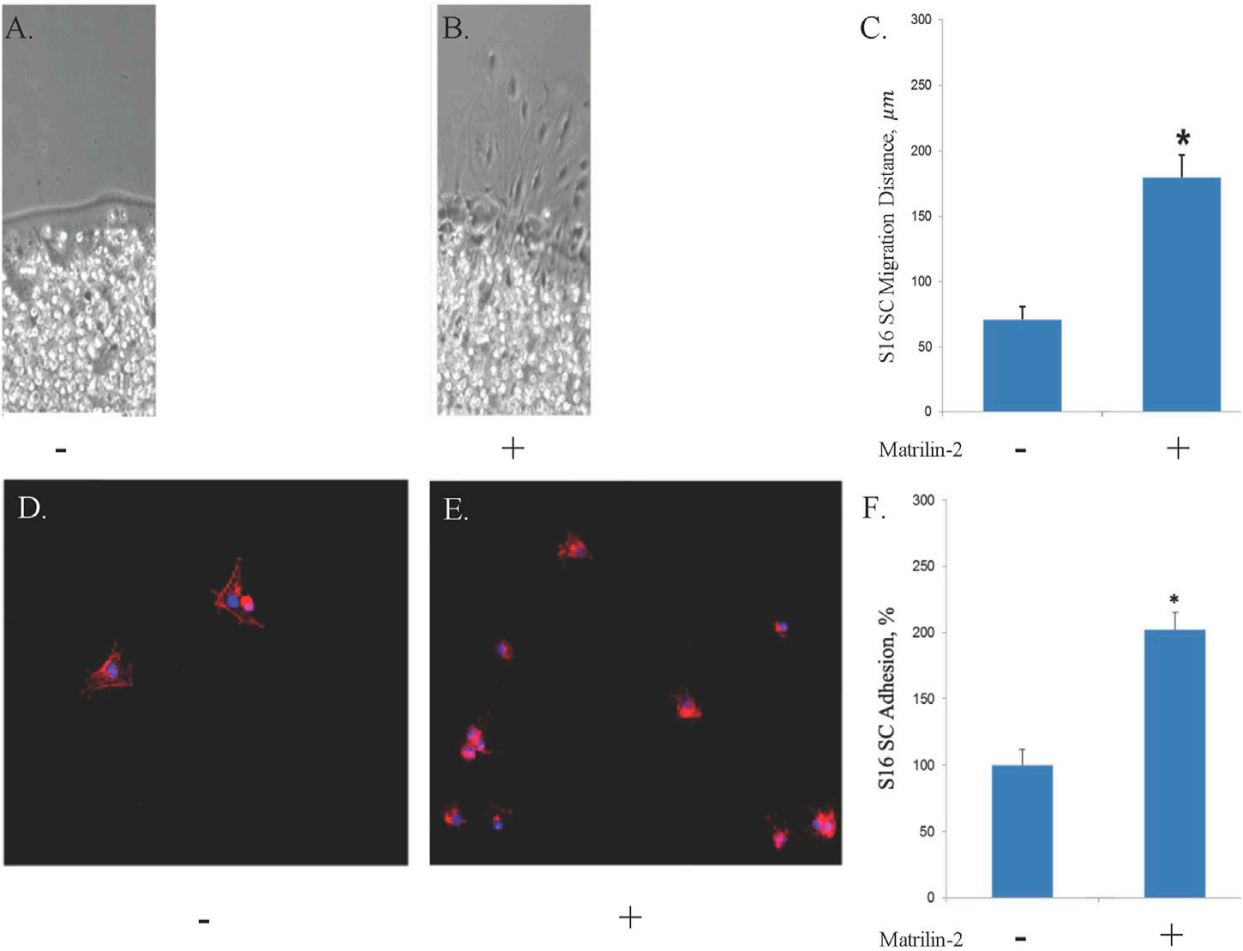
A-C, S16 SC agarose drop migration assay visualized by phase-contrast microscopy. Tissue glass coverslips coated with **(A)** ribonuclease-free water or **(B)** matrilin-2. Scale bar: 20 μm. **(C)** Quantification of migration distances noting significantly increased migration atop matrilin-2 compared to water. * denotes statistical significance of *p* < 0.0001. D-F, Fluorescent microscopy of Schwann cell adherence on tissue glass covered with **(D)** ribonuclease-free water and **(E)** matrilin-2. **(F)** Fluorescent profile denoting cellular presence was measured with Matrilin-2 compared against water noting a significantly greater presence of fluorescence and cellular adherence with matrilin-2. * denotes statistical significance of *p* < 0.01.

To determine the suitability of matrilin-2 as a bioactive component of the scaffold for nerve repair, we quantified its effect on Schwann Cell (SC) migration and adhesion. Agarose drop migration assay demonstrated a significantly greater migration of SCs atop matrilin-2 compared to no-matrilin-2 (Figures 2A,B). There was a significant increase in SC migration distance (180 ± 20 μm) atop matrilin-2 compared to no-matrilin-2 (0 μm) (*p* < 0.0001) (Figure 2C). SC adhesion was also significantly improved when incubated with matrilin-2 compared to no-matrilin-2 (Figures 2D,E). Quantification of adhered cells demonstrated a significantly greater adhesion profile on matrilin-2 compared to no-matrilin-2 (*p* < 0.001) (Figure 2F).

### Matrilin-2/chitosan Scaffold induced SC migration within the collagen conduit

**FIGURE 3:**
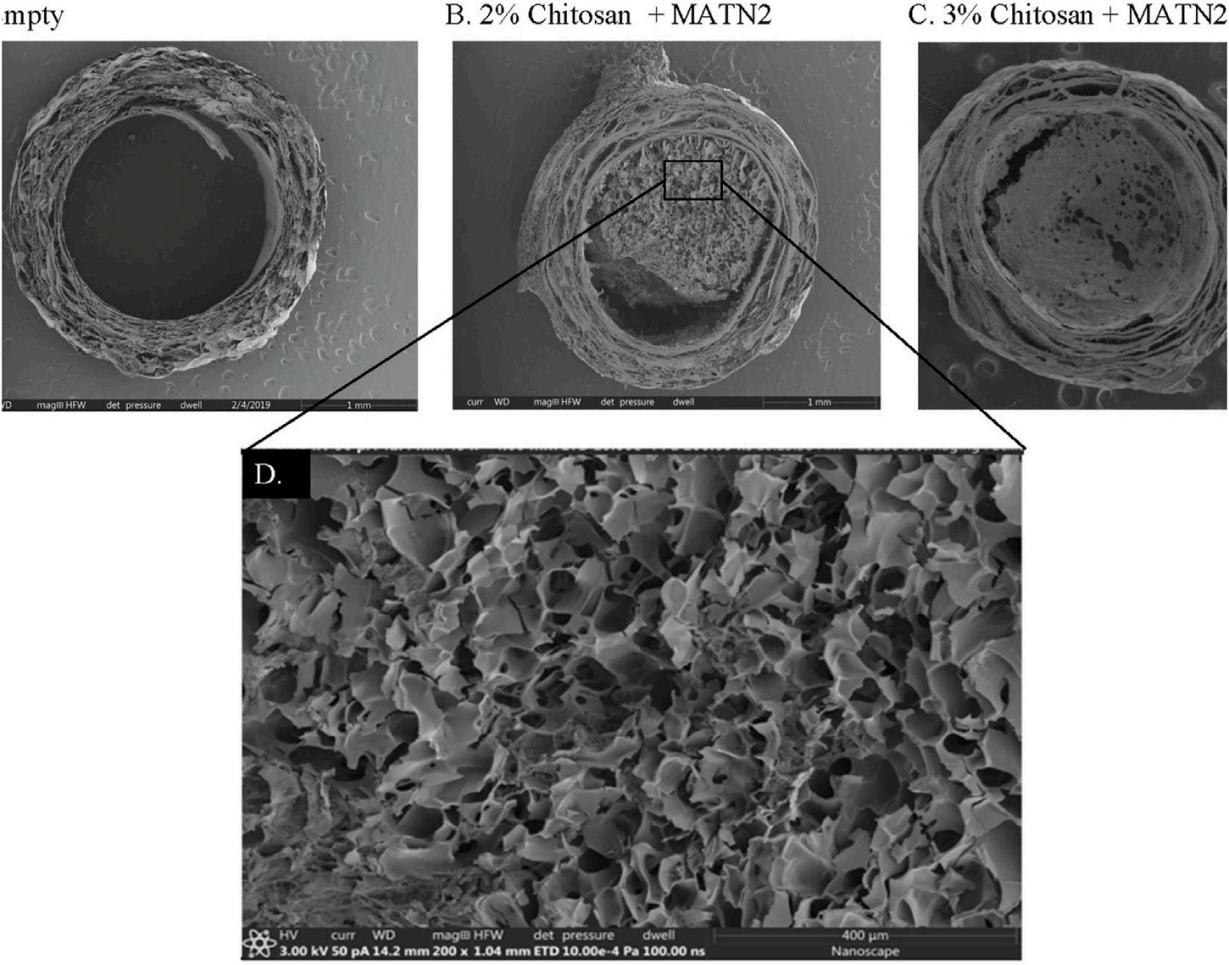
Scanning electron microscopy of **(A)** empty collagen conduit, **(B)** collagen conduit filled with matrilin-2 and 2% chitosan, and **(C)** collagen conduit filled with matrilin-2 and 3% chitosan. **(D)** Magnification of the matrilin-2 and 2% chitosan scaffold detailing porous structures measuring around 50 μm.

**FIGURE 4:**
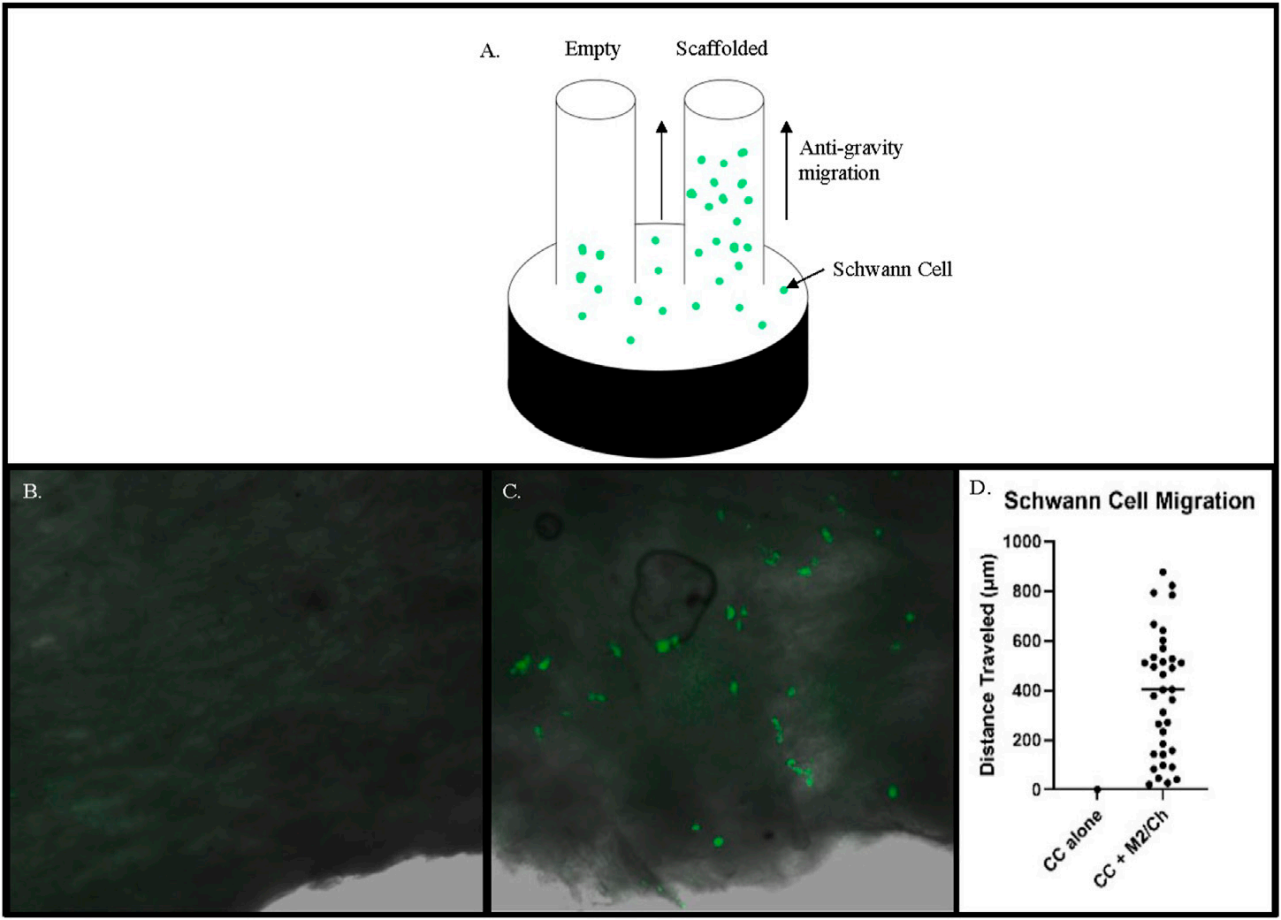
**(A)** Schematic of a modified capillary migration study that demonstrated negligible upward migration of Schwann cells in **(B)** empty collagen conduit (CC) while **(C)** Matrilin-2/Chitosan scaffold (M2/Ch) demonstrated migration of fluorescently labeled Schwann cells. **(D)** Quantification of distances traveled noted an average distance of 383.43 μm.

To test different formulations of the matrilin-2/chitosan scaffold within the collagen conduit, we performed scanning electron microscopy (SEM) imaging. A collagen conduit by itself presented a cross-sectional internal hollow tubular structure (Figure 3A). Matrilin-2 with 2% chitosan formed an internal scaffold with porous structures (Figure 3B). The pore sizes measured around 50 μm (Figure 3D). In contrast, matrilin-2 with 3% chitosan formed an amorphous scaffold without regular-sized pores (Figure 3C). The scaffold composed of matrilin-2 with 2% chitosan, referred to as matrilin-2/chitosan hereinafter was chosen for testing its biological activity. The matrilin-2/chitosan scaffold within the collagen conduit stimulated upward migration of fluorescently labeled SCs within the collagen conduit after 24h incubation, while a collagen conduit without the scaffold failed to do so (Figures 4A–C). The average upward migration distance in the scaffold was 383.43 μm (Figure 4D).

### Matrilin-2/K-chitosan scaffold induced DRG adhesion and axonal outgrowth

**FIGURE 5:**
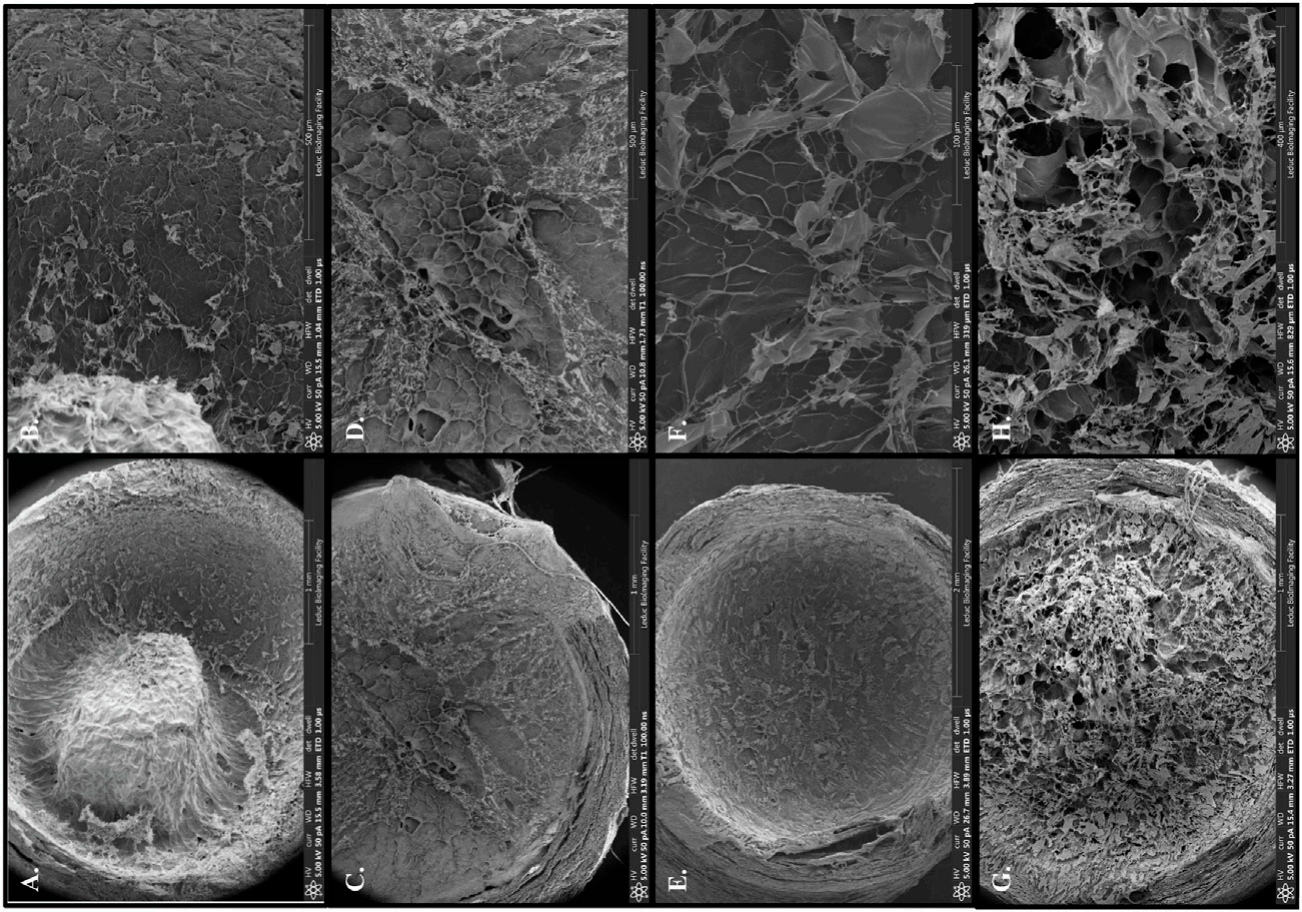
Scanning electron microscopy with overview of collagen conduit and scaffold components: **(A)** 2% chitosan, **(C)** matrilin-2/chitosan, **(E)** 2% chitosan modified with lysine (K-chitosan), **(G)** matrilin-2/K-chitosan. Scanning electron microscopy with focus on porosity of varying scaffold structures and components: **(B)** 2% chitosan with a dense and gapped matrix, **(D)** matrilin-2/chitosan with a more porous matrix, **(F)** K-chitosan with a dense and less gapped matrix, and **(H)** matrilin-2/K-chitosan a more 3D porous network.

To further improve matrilin-2 and chitosan interaction in the scaffold, we chemically modified chitosan with lysine (Figure 1B). Lysine is an important residue to mediate ECM crosslinking (Mäki et al., 2005). Scanning electron microscopy showed an irregular, dense, and gapped matrix with chitosan alone (Figures 5A,B). In matrilin-2/chitosan scaffold, a more porous structure was formed (Figures 5C,D). Chitosan modified with lysine (K-chitosan) presented a 2D cross-sectional architecture network (Figure 5 E, F). In contrast, matrilin-2/K-chitosan presented a 3D porous network structure with notable porosity and depth (Figures 5G,H). Such 3D porous structures are consistent with the natural channels noted in peripheral nerve structures (Jenq et al., 1987).

**FIGURE 6:**
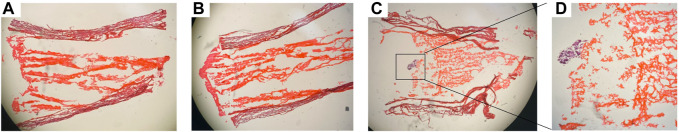
H&E staining of conduit demonstrating **(A)** collagen conduit with chitosan scaffold, **(B)** collagen conduit matrilin-2/chitosan scaffold, **(C)** collagen conduit with matrilin-2/K-chitosan scaffold with dorsal root ganglion (DRG) [black box]. **(D)** DRG at higher power adhere to matrilin-2/K-chitosan scaffold within collagen conduit.

To determine whether the matrilin-2/K-chitosan scaffold facilitates growth of DRGs in the collagen conduit, we first evaluated DRG adherence on the cross-sectional surface of the scaffold. H&E histological analysis was performed on the cross sections of the collagen conduit with and without the scaffold (Figure 6). The scaffold with chitosan only presented a dense structure without DRG adherence (Figure 6A). The scaffold with matrilin-2/chitosan demonstrated a more porous architecture however no DRG adhesion (Figure 6B). The scaffold with matrilin-2/K-chitosan demonstrated the most porous structure and a multi-channel architecture (Figures 6C,D). It facilitated the adhesion of the DRG to the matrilin-2/K-chitosan scaffold (Figure 6D).

**FIGURE 7:**
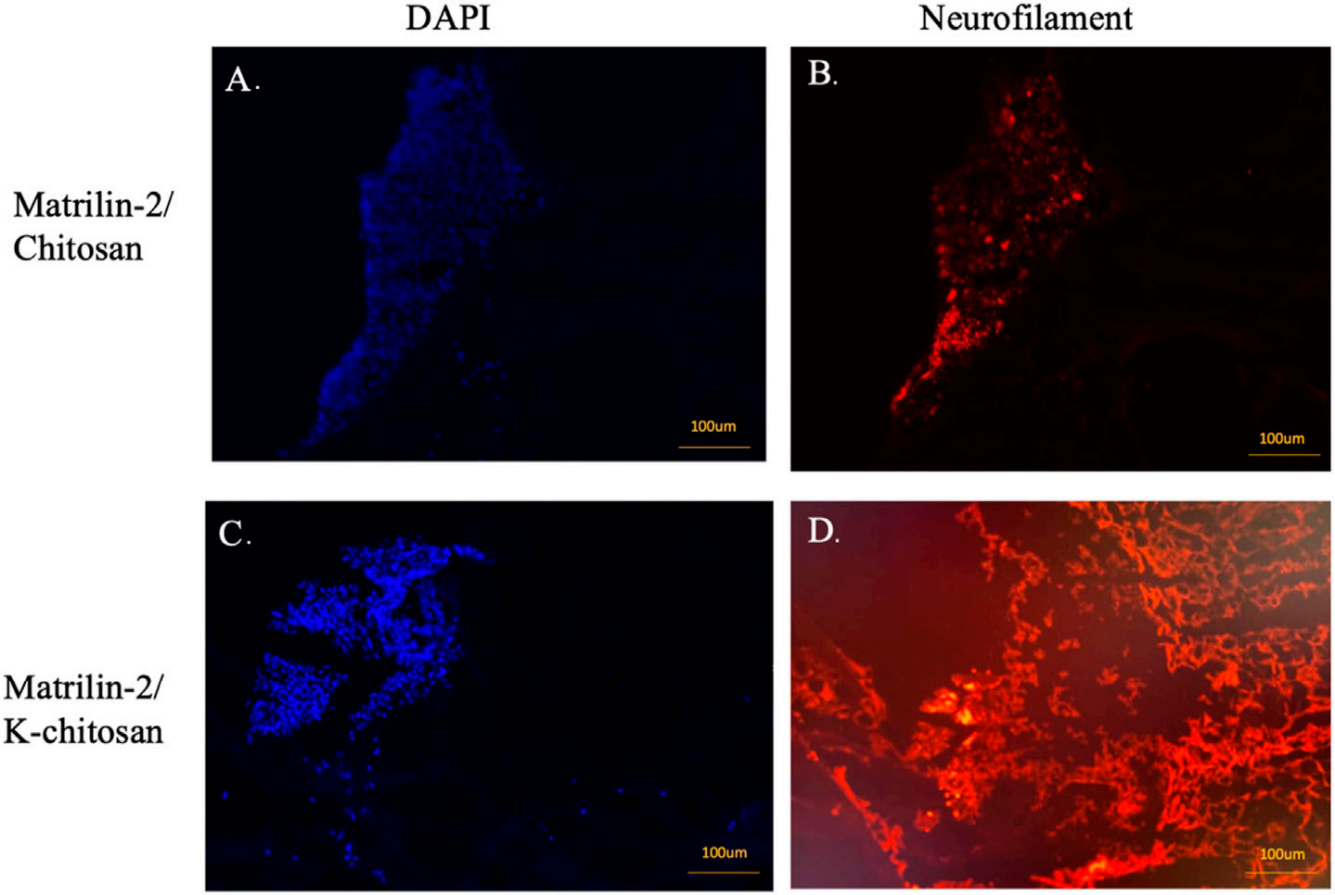
Immunofluorescent imaging of matrilin-2/chitosan and matrilin-2/K-chitosan scaffold. **(A)** DAPI staining of neuronal cells adhered to end of conduit with matrilin-2/chitosan scaffold and **(B)** neurofilament staining of matrilin-2/chitosan demonstrating minimal axonal outgrowth from neuronal cells seen of same DAPI field. **(C)** DAPI staining of cluster of neuronal cells adhered to the end of conduit and matrilin-2/K-chitosan scaffold and **(D)** neurofilament staining of same DAPI field demonstrating axonal outgrowth. Scale marked at 100uM.

To evaluate axonal outgrowth in the scaffolds, we performed immunofluorescence analysis with an antibody against neurofilament. The presence of neuronal cells in the scaffolds was detected by the DAPI nuclear staining. There was no DAPI staining in the chitosan-alone scaffold (data not shown). Although neuronal cells were observed in the matrilin-2/chitosan scaffold in the collagen conduit (Figure 7A), only residual neurofilament staining was observed (Figure 7B). In contrast, a greater number of neuronal cells comprising the DRG was observed in the matrilin-2/K-chitosan scaffold (Figure 7C). Numerous neurofilaments representing robust axonal outgrowth were observed in the matrilin-2/K-chitosan scaffold (Figure 7D).

## Discussion

In this study, we developed a novel 3D matrilin-2/K-chitosan porous scaffold for promoting axonal regeneration. Currently, commercially available, hollow collagen conduits are often used in clinical practice to assist with short gap distances. We demonstrated a significant superiority of the 3D porous matrilin-2/K-chitosan scaffold compared to not only the hollow collagen conduit, but also a scaffold comprising chitosan alone, or chitosan with matrilin-2. Our data indicated that the matrilin-2/K-chitosan scaffold forms a 3D porous network that is not only favorable to peripheral nerve regeneration structurally, but also induces Schwann cell migration, DRG neuronal cell adhesion, and axonal outgrowth biologically.

First, we identified chitosan as an optimal biomaterial for engineering a scaffold to stabilize matrilin-2 through materials analysis. Chitosan is a derivative of chitin, a natural polymer that makes up the structure of an invertebrate exoskeleton (Yi et al., 2018). The chitin is modified to contain a positively charged amine group on the oligosaccharide chain during a deacetylation reaction to create chitosan. The positive charge of the chitosan has been shown to enable cell adhesion and migration. In addition, chitosan has been demonstrated to be biocompatible and biodegradable and has been utilized to create nerve conduits (Yi et al., 2018)^,^ (Neubrech et al., 2018). Chitosan degradation products have been noted to improve upon peripheral nerve regeneration through stimulating SC proliferation and inducing macrophage infiltration (Wang et al., 2016; Zhao et al., 2017). However, chitosan lacks the ability to hold a cross-sectional structure without a crosslinker between its oligo-chains. With this understanding, matrilin-2 was utilized to successfully integrate and stabilize chitosan to create a porous multi-channel cross-sectional architecture when applied to a collagen conduit. We found that chitosan not only has material properties similar to those of the native nerve, but its chemical structure also allowed conjugating with matrilin-2 to form a scaffold. Constructing scaffolds with various concentrations of chitosan demonstrated that the 2% chitosan scaffold presented the best porous microarchitecture with matrilin-2.

Second, matrilin-2 was chosen to be the second important component of the scaffold based on its structural as well as biological activities. We sought to utilize the native presence of matrilin-2 in the neuronal tissues with its biological cues and structural interactions with the ECM and SCs. These properties are important for forming a biomimetic scaffold with not only a porous microarchitecture allowing nerve growth, but also the capability of enhancing SC cellular adhesion and migration and promoting axonal regeneration (Malin et al., 2009) Indeed, matrilin-2 facilitated greater SC migration and adhesion *in vitro*. Such results were consistent with prior literature showing matrilin-2 enhanced SC migration and adhesion greater than fibronectin or laminin (Malin et al., 2009). Matrilin-2 also increased axonal outgrowth from DRGs (Malin et al., 2009). Structurally, matrilin-2 serves as a hinge molecule in the ECM by forming an oligomeric structure (Klatt et al., 2011). Matrilin forms not only a filamentous structure by itself (Piecha et al., 1999), but also a filamentous network by interacting with other ECM ligands including collagens and proteoglycans (Wang et al., 2014). The broad interaction of matrilin-2 allows it act not only as a mediator between ECM proteins, but also induce a chemotactic property for SC migration. Further establishment of cellular interactions with matrilin-2 within the microenvironment of peripheral nerve regeneration will help to better elucidate its mediating role between injury response and induction of regeneration.

Although the matrilin-2/chitosan scaffold induced SC migration against gravity in the collagen conduit, it failed to facilitate DRG adhesion or axonal outgrowth during the 3D organotypic assay. Thus, we focused on the creation of a 3D biomimetic extracellular matrix environment to improve the efficiency of cellular adhesion and migration during peripheral nerve regeneration. The modification of chitosan with lysine provides a greater interaction of matrilin-2 with chitosan by adding a free amine group that is less sterically hindered. Lysine has further been used to augment hydrogels to promote neurite outgrowth by improving bioactivity through delivery of positive charges (Cai et al., 2012a; 2012b). Indeed, we observed a notably increased porosity and depth of the 3D network in the matrilin-2/K-chitosan scaffold than that without lysine modification. Such porous structure mimics the native channels observed in native peripheral nerves (Jenq et al., 1987). Prior research of lysine has also demonstrated that its presence creates a hydrophilic surface and surface charge that improves the environment for cellular adhesion (Wang et al., 2018). Differences in nerve cell affinity were observed through various binding patterns of lysine with chitosan (Cheng et al., 2004). Lysine also improved the affinity, differentiation, and growth of neurons. Our study demonstrated that the 3D matrilin-2/K-chitosan scaffold not only presented an optimal porous cross-sectional architecture, but also supported DRG affinity and induced axonal outgrowth.

A limitation of this study is that the novel scaffolds were tested with SCs *in vitro* and with DRG *ex-vivo*. We plan to analyze the scaffold with an *in-vivo* rat sciatic nerve model to further understand its impact on peripheral nerve regeneration compared to an empty conduit and autograft. Ultimately, this work serves as a template for establishing a scaffold capable of integrating crucial extracellular matrix proteins, chemokines, and growth proteins. It creates a biomimetic microenvironment that improves the adhesion and infiltration of SCs through the site of bridging during nerve regeneration. By optimizing SC behavior towards a reparative phenotype following nerve repair, the environment for axonal regeneration is better established and maintained, thus allowing for a more rapid recovery from potentially devastating injuries to peripheral nerves.

## Conclusion

The delivery of matrilin-2, a crucial extracellular matrix protein upregulated during nerve injury, to assist in creating a biomimetic environment to help bridge critical nerve gaps has yet to be thoroughly explored, leaving much of its biomedical properties and potential untapped. Integrating matrilin-2 into chitosan modified with lysine scaffold produced a porous internal structure that promoted Schwann cell migration DRG adhesion and axonal outgrowth. Such findings demonstrate the potential of using chitosan and matrilin-2 to engineer a 3D microenvironment to improve upon SC response following injury. Ultimately, our scientific premise lies in generating nerve grafts that not only have the multi-channel microarchitecture of nerve, but are bioactive in their interaction with SC adhesion, migration, and reparative activity. Such a novel scaffold supports the pathway for axonal outgrowth and becomes a promising, reliable, and readily available therapeutic for nerve repair.

## Data Availability

The raw data supporting the conclusion of this article will be made available by the authors, without undue reservation.
