# Supplementary figures and images for: Matrilin-2 within a three-dimensional lysine-modified chitosan porous scaffold enhances Schwann cell migration and axonal outgrowth for peripheral nerve regeneration
Source: Front Bioeng Biotechnol. 2023 May 4;11:1142610. doi: 10.3389/fbioe.2023.1142610 (PMC10201561; doi:10.3389/fbioe.2023.1142610)

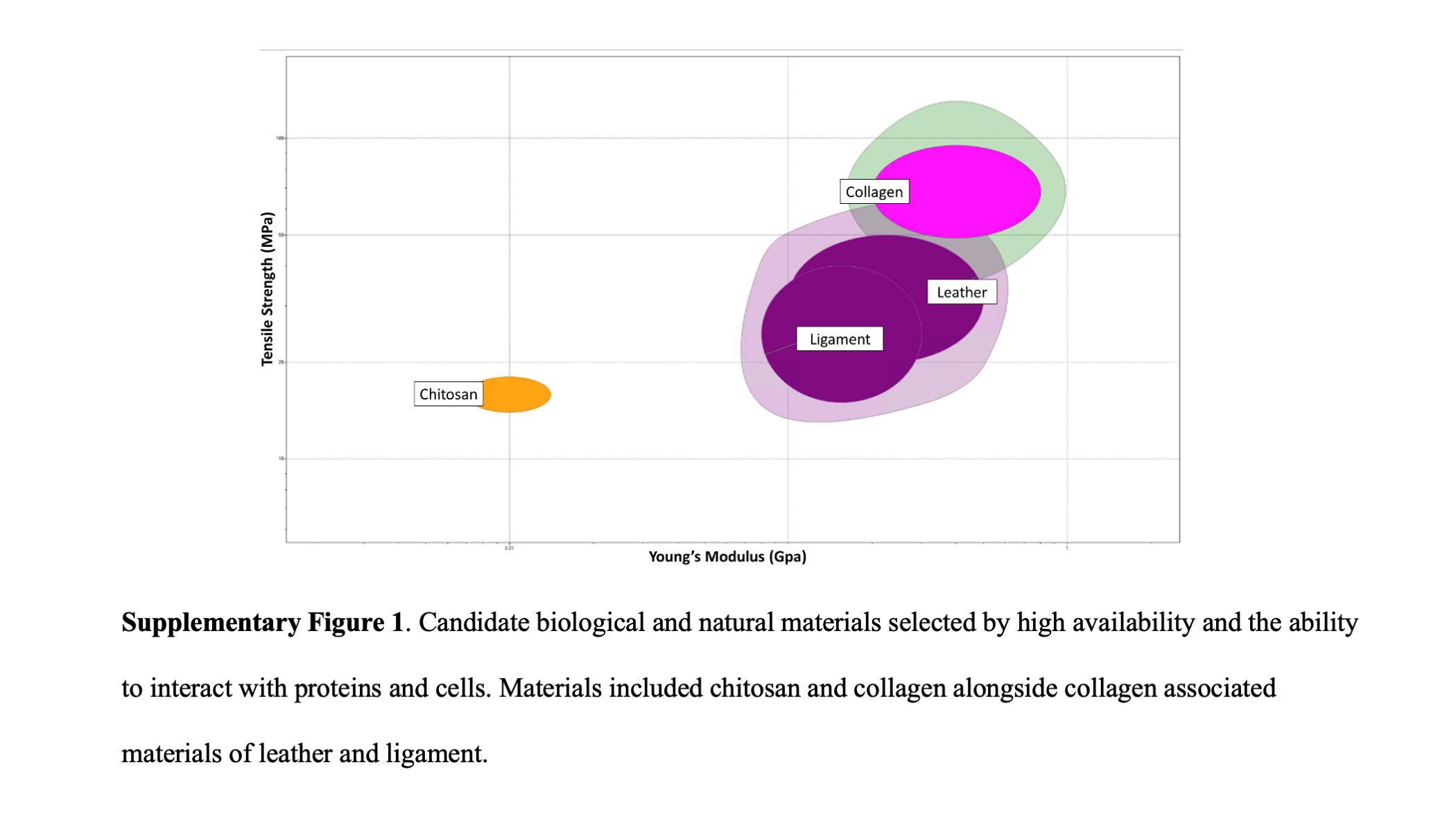

Supplement: Supplementary file 1 [file Image1.TIFF]
